# Incorrect Citations Give Unfair Credit to Review Authors in Ecology Journals

**DOI:** 10.1371/journal.pone.0081871

**Published:** 2013-12-11

**Authors:** Mariana C. Teixeira, Sidinei M. Thomaz, Thaisa S. Michelan, Roger P. Mormul, Thamis Meurer, José Vitor B. Fasolli, Márcio J. Silveira

**Affiliations:** Universidade Estadual de Maringá, Nupélia, Maringá-PR, Brazil; Max Planck Society, Germany

## Abstract

The number of citations that papers receive has become significant in measuring researchers' scientific productivity, and such measurements are important when one seeks career opportunities and research funding. Skewed citation practices can thus have profound effects on academic careers. We investigated (i) how frequently authors misinterpret original information and (ii) how frequently authors inappropriately cite reviews instead of the articles upon which the reviews are based. To reach this aim, we carried a survey of ecology journals indexed in the Web of Science and assessed the appropriateness of citations of review papers. Reviews were significantly more often cited than regular articles. In addition, 22% of citations were inaccurate, and another 15% unfairly gave credit to the review authors for other scientists' ideas. These practices should be stopped, mainly through more open discussion among mentors, researchers and students.

## Introduction

The need to evaluate scientific impact has led to the development of a myriad of metrics such as the mean number of citations per paper, the total number of citations of a given author [1] and the H index [2]. An advantage of these indices is that they evaluate directly the author's work instead of using the impact factor (IF) of the journals where scientists publish. Therefore, scientists seek out citations but also question if they express impartially one's scientific contribution [3]. Some biases associated the number of citations (e.g., author gender, number of authors, length of articles and language) have already been investigated in ecology publications [4], [5]. Our concern is that the accuracy of citations is rarely, if ever, checked by reviewers and editors and might be deceptive. Some skewed citation habits have been detected, such as excessive self-citation [6], preference for open access journals [7] and incorrect citations [8]–[10].

Review papers are often widely cited and are considered to be general indicators of quality in scientific production [11]. Such papers are supposed to “establish new benchmarks in the field, define directions for future research, contribute to fundamental understanding of ecological principles, and whenever possible, derive principles for ecological management in its broadest sense,” according to the journal *Ecological Monographs* [12]. However, they can also be seen as assortments of information and literature that can easily impel bad citation practices, such as “empty references” [13]. These references can be defined as those “that do not contain any original evidence for the phenomenon under investigation, but strictly refer to other studies to substantiate their claim” [13]. Because empty references may provide readers with inaccurate information and do not recognize the author who originally proposed an idea [14], they may be considered misleading and unethical.

Here, we surveyed standard research papers (hereafter “articles”) in the field of ecology to assess citation habits and possible biases and misconduct among authors in their citation of review papers. First, we investigated whether reviews are cited more often than articles by ecology scientists. Second, we assessed the frequencies of the misinterpretation of the concepts and ideas of the reviews (see [13]) and of citations that fit the “lazy author syndrome” (*sensu* [14]), hereafter “misinterpreted” and “lazy” citations, respectively. The latter type of inappropriate citation occurs when an author attributes findings to the author(s) of the latest review on the matter instead of giving the proper credit to the scientist(s) who first described those findings [14].

## Methods

Our citation analyses were based on papers classified as reviews. We first used the classification given in the Web of Science database to search for reviews. However, because classification of reviews in this database can be inaccurate, we read each paper carefully and kept for further analyses only those ones that were really reviews.

**Figure 1:**
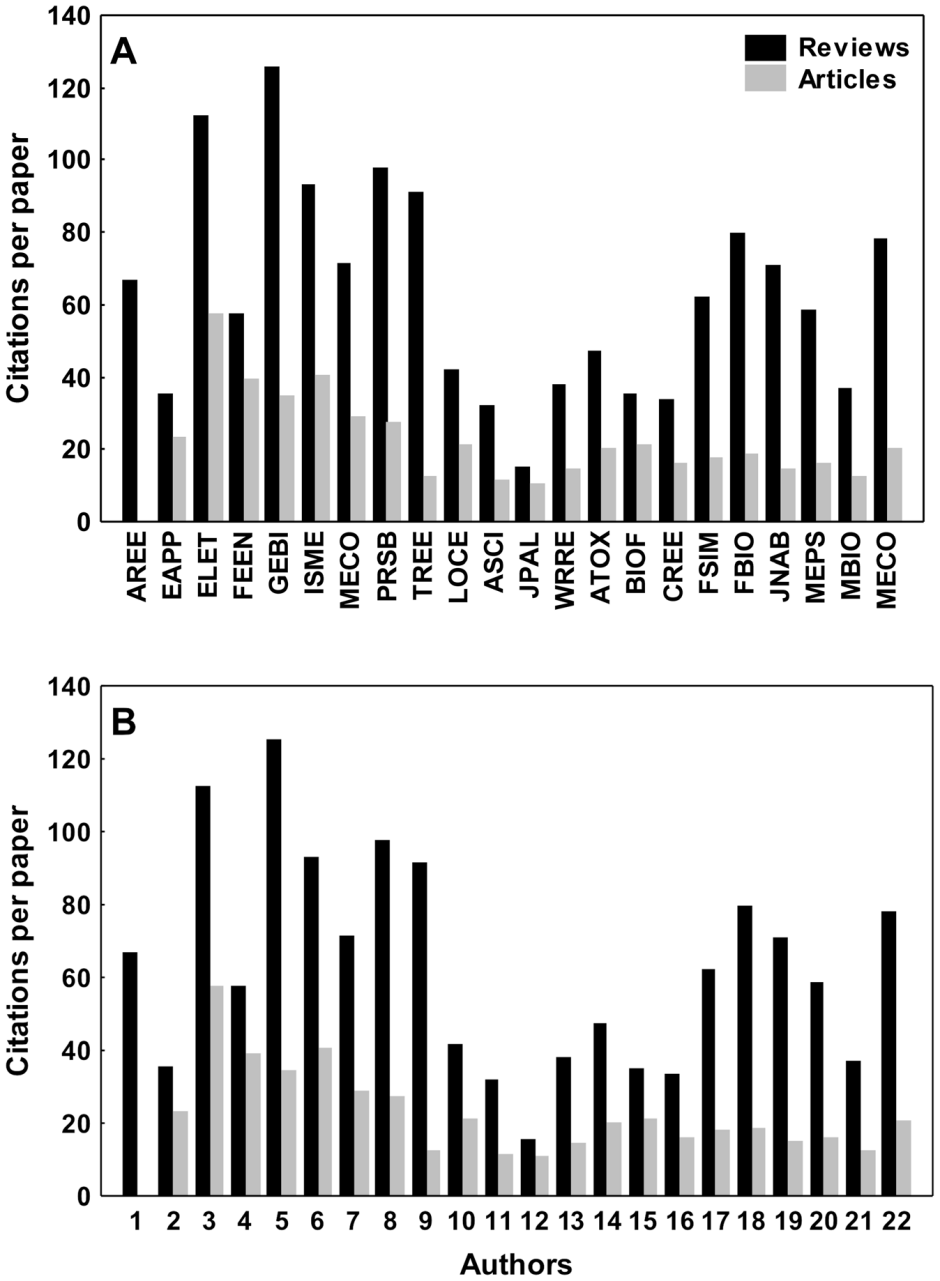
Mean number of citations per paper for reviews and articles of journals in the areas of ecology, limnology and marine and freshwater biology (A) and for authors of the analyzed reviews (B). Annual Review of Ecology Evolution and Systematics (AREE; n_ART_ = 0; n_REV_ = 33); Ecological Applications (EAPP; n_ART_ = 194; n_REV_ = 8); Ecology Letters (ELET; n_ART_ = 90; n_REV_ = 14); Frontiers in Ecology and the Environment (FEEN; n_ART_ = 35; n_REV_ = 19); Global Ecology & Biogeography (GEBI; n_ART_ = 74; n_REV_ = 3); ISME Journal (ISME; n_ART_ = 63; n_REV_ = 3); Molecular Ecology (MECO; n_ART_ = 372; n_REV_ = 23); Proceedings of the Royal Society B-Biological Sciences (PRSB; n_ART_ = 361; n_REV_ = 9); Trends in Ecology & Evolution (TREE; n_ART_ = 19; n_REV_ = 70); Limnology & Oceanography (LOCE; n_ART_ = 236; n_REV_ = 3); Aquatic Sciences (ASCI; n_ART_ = 46; n_REV_ = 1); Journal of Paleolimnology (JPAL; n_ART_ = 67; n_REV_ = 5); Water Resources Research (WRRE; n_ART_ = 406; n_REV_ = 6); Aquatic Toxicology (ATOX; n_ART_ = 164; n_REV_ = 3); Biofouling (BIOF; n_ART_ = 36; n_REV_ = 4); Coral Reefs (CREE; n_ART_ = 84; n_REV_ = 5); Fish & Shellfish Immunology (FSIM; n_ART_ = 190; n_REV_ = 3); Freshwater Biology (FBIO; n_ART_ = 184; n_REV_ = 4); Journal of the North American Benthological Society (JNAB; n_ART_ = 61; n_REV_ = 1); Marine Ecology Progress Series (MEPS; n_ART_ = 628; n_REV_ = 10); Marine Biotechnology (MBIO; n_ART_ = 60; n_REV_ = 8); Microbial Ecology (MECO; n_ART_ = 140; n_REV_ = 2).

To assess whether reviews are more often cited than articles, we selected among the top-IF journals (year 2011) that published reviews within the Thomson Reuters Web of Knowledge database. Using this criterion, we selected nine journals in the field of ecology (IF from 5.41 to 17.46), nine in the field of marine and freshwater biology (IF from 2.71 to 4.42), and four journals in the field of limnology (IF from 1.89 to 3.41) (see caption of Fig. 1), and we compared the number of citations per paper between reviews and articles. Because the number of reviews and articles within each journal are not the same, we considered each category and then divided the total number of citations by the number of papers for each journal (see Fig. 1). The results of this analysis provide information about the IF inflation of journals that publish reviews. To estimate the citations of reviews and articles by individual authors, we used the previously selected 22 reviews (see below) and checked the citations that these authors had received for their 2007 paper up to April 2013, distinguishing between reviews and articles. These results indicate the inflation of individual research impact. In both cases, we used a *t*-test to assess differences between citations of reviews and of articles.

We used the most cited review published in 2007 from each of the 22 selected journals, according to the above criterion, to assess the percentage of misinterpreted and lazy citations. To complete this task, we examined the ten most recent (October 2012) articles that cited each of the 22 selected reviews. We analyzed a total of 217 articles; we excluded one article that was not written in English and two other articles that could not be accessed. The reviews were completely read to clarify their main message(s), and the citing articles were screened for citations of the examined reviews, which were scored as correct, misinterpreted or lazy. Citations that did not show coherent or clear relation to the ideas presented in the review were deemed misinterpreted. Although the borderline between acceptable and lazy review citations is somewhat blurred, we deemed lazy to be those citations that recalled an idea that was not the review author's but was originally presented in another article cited by the review's author. During our evaluation of articles, we discussed together whenever there were doubts of whether the citation was misinterpreted or lazy, or not. Based on these analyses, we calculated the percentages of misinterpreted, lazy and correct citations and an article error rate by dividing the number of articles with at least one misinterpreted or lazy citation by the total number of articles examined.

Finally, to check whether a journal's impact factor predicts bad citation practice, we analyzed the relationship between the occurrence of misinterpreted and lazy citations and the journal's impact factor using a logistic regression [15]. We used the presence (1) or absence (0) of lazy or misinterpreted citations as the categorical response and the journal's impact factor as the predictor variable.

## Results

We found a total of 223 reviews and 1242 articles in the top 10 journals by IF in the field of ecology published in 2007 (see Fig. 1 caption). Reviews received significantly more citations than articles published in the same journals (t = 6.10; *p*<0.001; Fig. 1a). Citations of all reviews published by the selected authors were also higher than citations received by their papers (t = 4.45; *p*<0.001; Fig. 1b).

We evaluated a total of 334 citations of reviews in the 217 articles examined, out of which 63% were correct, 15% were misinterpreted and 22% were lazy. The article error rate was 41%. Clear examples of misinterpreted and lazy citations were found referencing the Rose and Caron review [16]. One article stated that Rose and Caron [16] demonstrated that the activation energy in phytoplankton populations should be 0.65 eV for respiration and 0.32 eV for photosynthesis. However, these numbers do not appear in the Rose and Caron review. We consider this a typical case of a misinterpreted citation. In another article, Rose and Caron [16] are cited as demonstrating that phytoplankton blooms might occur in places where extremely low temperatures persist year round. However, in Rose and Caron [16], this information is credited to Caron et al. [17]; this case was identified as a typical lazy citation.

The logistic regression did not show a relationship between the numbers of misinterpreted or lazy citations and a journal's IF (Table 1) because the probability of occurrence of each practice did not change significantly with increasing IF (Table 1).

**Table 1:** Results of the logistic regression using the presence of misinterpreted and lazy citations as dependent variables and the journal impact factor (IF) as the predictor.

	Estimate	X^2^	Odds ratio	McFadden's rho^2^	P
Misinterpreted citations					
IF	0.04	1.03	1.04	0.005	0.31
Lazy citations					
IF	0.01	0.063	1.01	0.0003	0.81

^2^ estimates the proportion of variation explained by a logistic regression model. McFaddens' rho

The odds ratio tests the odds of misinterpreted and lazy citations occurrence according to journal impact factor.

## Discussion

Our results show that reviews receive more citations than articles, most likely due to their broadness and ease of information access, but more than one-third of these citations were inaccurate. Roughly one out of every five ecology articles that cite reviews did not give proper credit to the authors who first had the idea and/or performed a certain investigation. The proportion of lazy citations we found was higher than those reported in other similar studies involving ecological publications [8], [10]. However, our data are specifically on the citation of review papers, whereas these two past publications refer to other types of improper citations and include all types of papers. Thus, our results suggest two outcomes: (i) journals publishing more reviews may have their IFs inflated by inaccurate and lazy citations and (ii) review authors have greater chances of being erroneously cited, while those authors who originally had an idea have a greater chance of being neglected when their articles are referred to in a review.

We would expect that inaccuracy of citations in terms of both misinterpreted and lazy citations would decrease with journal IF because these bad citation practices should not occur among authors who submit to these journals or be neglected by their editors and reviewers, who theoretically are highly qualified scientists. Indeed, Drake et al. [10] showed an increase in inaccurate citations with decreasing journal IF, and they interpreted this finding as evidence of better quality for journals with higher IFs. In contrast, our logistic regression did not detect any association of the frequency of misinterpreted and lazy citations with journal IF, indicating that at least for citations of reviews, the journal quality as measured by its IF does not matter. However, it should be noted that our dataset included only high-IF journals (IF>1.89), while Drake et al. [10] found a 51% frequency of citation inaccuracy in low-IF journals (IF<1.0). In addition, the range of IFs in our dataset was unbalanced, with 86% of journals with IFs lower than 5, 12% between 5 and 10 and only 2% higher than 10. Thus, the frequency of misinterpreted citations may be underestimated.

Although our findings concerning lazy citations allude to ethical problems, they certainly indicate that bad citation practices have consequences for scientists' career success. Because indices based on the number of citations are attaining great importance in some countries (e.g., [18]), lazy citations have negative effects on authors whose articles are not cited and introduce positive biases toward authors whose reviews are cited improperly. However, we were not able to quantify how negative this impact would be to the neglected authors. In addition, our results showed a great variation of individual citations attributed to reviews or articles (see Fig. 1b), which indicates that some authors have their individual impact (e.g., any measurement that takes individual number of citations) more inflated by lazy citations than others.

Roughly one out of six articles that cited a review did not relay the review's original ideas properly, misinterpreting them. This is a much lower rate than the one found by Drake et al. [10]: 37.9% of citations were partially supported or totally unsupported considering journals of all IFs. Misinterpretation of a referenced paper can be considered one of the most damaging violations of academic referencing [13]. It is not fair to the cited author and conveys wrong information to readers, who may disseminate the erroneous reference (see [10]). Lortie et al. [19] showed that in the area of ecology and evolutionary biology, citation choice is not associated with data consistency on a certain hypothesis. A typical example is the paper by Wilcove et al. [20], which became a classical paper in invasion biology. Hundreds of papers cite this paper to highlight that invasions are the second greatest threat to native species, although its limitations and biased information do not consistently support this conclusion [21]. Considering the poor citation practices found in our and other investigations [9]–[10], [13], we suspect that such cases might be more widespread in science than previously expected.

In summary, our data suggest that journals' IFs and review authors' individual impact can be unfairly inflated by misinterpreted and lazy citations. Our data also show that even high-IF journals disseminate inaccurate information. With so many indications of poor citation practices, we think that care should be taken to reduce them and improve reliability in science. For example, co-authors of a manuscript should be critical about papers cited by their co-authors, and verification practices like this should not be seen as mistrustful [22]. Other attitudes depend more on mentors, who should discuss more with lab members, [22] and on teachers and advisors, who should discuss with graduate (and even undergraduate) students [10] more openly about these (and other) scientific misconducts. Although no single proposal is capable of eliminating poor citation practices, we think that a combination of attitudes (like those mentioned above) could contribute to reducing them.
