# Correction: Incorrect Citations Give Unfair Credit to Review Authors in Ecology Journals

**DOI:** 10.1371/annotation/c97fe584-a2c8-47d1-b051-a61c1606da48

**Published:** 2013-12-30

**Authors:** Mariana C. Teixeira, Sidinei M. Thomaz, Thaisa S. Michelan, Roger P. Mormul, Thamis Meurer, José Vitor B. Fasoli, Márcio J. Silveira

The name of the sixth author was spelled incorrectly. The correct name is: José Vitor B. Fasoli. 

